# High-definition endoscopy with digital chromoendoscopy for histologic prediction of distal colorectal polyps

**DOI:** 10.1186/s12876-015-0374-3

**Published:** 2015-10-22

**Authors:** Timo Rath, Gian E. Tontini, Andreas Nägel, Michael Vieth, Steffen Zopf, Claudia Günther, Arthur Hoffman, Markus F. Neurath, Helmut Neumann

**Affiliations:** 1 Interdisciplinary Endoscopy, Department of Medicine I, University Hospital Erlangen, Ulmenweg 18, 91054 Erlangen, Germany; 2 Institute of Pathology, Klinikum Bayreuth, Bayreuth, Germany; 3 Department of Medicine II, HSK Wiesbaden, Wiesbaden, Germany

**Keywords:** Adenomas, Colorectal cancer, Gastrointestinal endoscopy, Histology, Polyps

## Abstract

**Background:**

Distal diminutive colorectal polyps are common and accurate endoscopic prediction of hyperplastic or adenomatous polyp histology could reduce procedural time, costs and potential risks associated with the resection. Within this study we assessed whether digital chromoendoscopy can accurately predict the histology of distal diminutive colorectal polyps according to the ASGE PIVI statement.

**Methods:**

In this prospective cohort study, 224 consecutive patients undergoing screening or surveillance colonoscopy were included. Real time histology of 121 diminutive distal colorectal polyps was evaluated using high-definition endoscopy with digital chromoendoscopy and the accuracy of predicting histology with digital chromoendoscopy was assessed.

**Results:**

The overall accuracy of digital chromoendoscopy for prediction of adenomatous polyp histology was 90.1 %. Sensitivity, specificity, positive and negative predictive values were 93.3, 88.7, 88.7, and 93.2 %, respectively. In high-confidence predictions, the accuracy increased to 96.3 % while sensitivity, specificity, positive and negative predictive values were calculated as 98.1, 94.4, 94.5, and 98.1 %, respectively. Surveillance intervals with digital chromoendoscopy were correctly predicted with >90 % accuracy.

**Conclusions:**

High-definition endoscopy in combination with digital chromoendoscopy allowed real-time *in vivo* prediction of distal colorectal polyp histology and is accurate enough to leave distal colorectal polyps in place without resection or to resect and discard them without pathologic assessment. This approach has the potential to reduce costs and risks associated with the redundant removal of diminutive colorectal polyps.

**Trial registration:**

ClinicalTrials NCT02217449.

## Background

Distal diminutive polyps (polyps ≤ 5 mm) are frequently observed in daily clinical routine and can be found in more than 50 % of the screening population [1, 2]. However, diminutive polyps have a very low prevalence of advanced histologic features [3, 4] and their cancer prevalence ranges between 0 and 0.08 % [5, 6]. Therefore, histology of diminutive polyps is mostly used to guide post-polypectomy surveillance. Since conventional white-light endoscopy cannot reliably distinguish histological features of colorectal polyps, it is standard practice to remove all polyps for histopathological evaluation. However, this routine pathologic evaluation of all resected diminutive polyps results in considerable costs to the health care system for the management of a group of lesions which eventually have only limited clinical significance. The annual up-front cost savings in the US of forgoing the pathologic assessment would exceed one billion dollars per year [7].

Recently, the American Society for Gastrointestinal Endoscopy (ASGE) proposed two new paradigms for the management of diminutive polyps in the so called PIVI-statement [8]. One paradigm describes endoscopic resection of colorectal polyps without submitting them for pathological assessment (“resect and discard”) while the other paradigm describes to leave diminutive hyperplastic rectosigmoid polyps in place without resection [8]. A prerequisite for both approaches is that an accurate real-time endoscopic assessment of the colorectal polyp histology could be made [8].

Within the recent years, dye-less chromoendoscopy techniques (DLC) are rapidly evolving allowing characterization of the tissue in real-time without the requirement of traditional dye-spraying. DLC includes optical (i.e. NBI, CBI) and digital (i.e. i-scan, FICE, SPIES) chromoendoscopy [9–12].

Recently, it was shown that NBI is reliable for *in vivo* prediction of distal polyp histology with a negative predictive value of ≥ 90 % [13–16]. Nonetheless, data on the prediction of distal polyp histology with digital chromoendoscopy are scarce to date. Here, we prospectively assessed the potential of digital chromoendoscopy for real-time *in vivo* prediction of distal diminutive polyp histology according to the requirements of the ASGE PIVI statement.

## Methods

### Study design, setting and patient recruitment

We conducted a prospective observational study of diminutive polyps within the distal colorectum identified during screening or surveillance colonoscopies at the Ludwig Demling Endoscopy Center of Excellence at the University Hospital Erlangen. The study was approved by the local ethics committee (Ethical Commitee of the Medical Faculty of the Friedrich-Alexander University Erlangen-Nuremberg) and conforms to the provisions of the Declaration of Helsinki. ClinicalTrials Registration number is NCT02217449. Written informed consent was obtained from all patients prior to the procedure. Patients with a history of IBD, poor bowel preparation, colectomy, anticoagulation or polyposis syndrome were excluded.

### Endoscopic and histological assessment of polyps

All colonoscopies were performed by a single experienced endoscopist. Polyps were defined as distal if located in the descending colon, the sigmoid colon, or the rectum. High-definition colonoscopes equipped with digital chromoendoscopy (Pentax, Tokyo, Japan) were used for all examinations. Upon visualization of a polyp in white-light, the location and size (as compared to open biopsy forceps or snare) were noted. Afterwards, i-scan was used to visualize and enhance the mucosal vascular pattern and the mucosal surface pattern of the polyp. The endoscopist made a real time assessment of each polyp according to size, shape, Paris classification [17] and surface characteristics including pit pattern and mucosal vascular pattern morphology, colour, and type of depression. Further, the endoscopist assigned a level of confidence (high or low) to each polyp [13, 16]. Afterwards, all polyps were resected using standard techniques and processed for pathological evaluation. Each polyp was assessed by an experienced GI pathologist blinded to the real time prediction of polyp histology. Finally, real time and histological results were compared.

### Variables and statistics

Assessing the sensitivity and negative predictive value of digital chromoendoscopy for prediction of adenomatous polyp histology according to the recent PIVI statement [8] was the primary study endpoint. The pathology report was used as a reference point for the validation of the endoscopic assessment. Accuracy, diagnostic sensitivity and specificity and negative and positive prediction were calculated using SPPS 21.0 and are presented according to the STARD guidelines [18]. To assess the ability of i-scan to predict post-polypectomy surveillance intervals, the intervals that would be recommended by endoscopic prediction were compared with those that would be recommended by pathologic assessment as recommended in European [19] and US guidelines [20]. For this study we set the probability for error (α) to 0.05 and the ß-error to 0.1 (reflecting a power of 0.90). For white light endoscopy, an expected accuracy of 74 % and for i-scan an expected accuracy of 90 % was assumed [21–23], resulting in a calculated sample size of 120 polyps. The intraobserver agreement was calculated by using the percentage agreement and the values of κ statistics: slight, ≤0.2; fair, 0.21–0.4; moderate, 0.41–0.6; substantial, 0.61–0.80; and almost perfect, 0.81–1.00.

## Results

### Study cohort

224 consecutive patients undergoing screening or surveillance colonoscopy were prospectively included in the study. Of these, 77 patients had a total of 121 distal colorectal polyps. Of the 121 polyps, 42 were located in the descending colon, 32 in the sigmoid colon and 47 polyps were located in the rectum. Clinical, demographic and histologic characteristics of the patients and polyps are summarized in Table 1. The median size of the polyps was 3 mm and all polyps studied were diminutive, thereby ≤ 5 mm in size. 63 polyps were nonadenomatous, of which almost all were hyperplastic by histology (62 out of 63) while 1 polyp was a leiomyoma. 47 % of all polyps showed adenomatous histology (57 out of 121). Of these adenomas, the vast majority (46 out of 57) were tubular while seven adenomas exhibited tubulovillous histology. Further, four polyps were sessile serrated adenomas (SSAs) on histology, all of which were located in the descending colon.

**Table 1:** Clinical characteristics of the patients and polyps

	Patient characteristics	
	Patients with distal colorectal polyps (*n* = 77)	Patients with rectosigmoid polyps (*n* = 59)
Sex, n (%)		
Male	49 (63.6)	37 (62.7)
Female	28 (36.4)	22 (37.3)
Age, y		
Mean ± SD	65.5 ± 14.4	61.9 ± 15.2
Median (range)	67 (22–83)	66 (22–83)
	Polyp characteristics	
	Polyps in the distal colorectum (*n* = 121)	Polyps in the rectosigmoid (*n* = 79)
Location, n (%)		
Descending colon	42 (34.7)	
Sigmoid	32 (26.5)	32 (40.5)
Rectum	47 (38.8)	47 (59.5)
Histology, n (%)		
Adenoma	57 (47.1)	29 (36.7)
Tubular	46 (38)	27 (34.2)
Tubulovillous	7 (5.8)	2 (2.5)
SSA	4 (3.3)	0 (0)
Hyperplastic	63 (52.1)	50 (63.3)
Other (Leiomyoma)	1 (0.8)	
Size, n (%)		
≤ 3 mm	75 (62)	54 (68.4)
4–5 mm	46 (38)	25 (31.6)
median (mean), mm	3 (3.3)	3 (3.2)

SSA, sessile serrated adenoma

### Diagnostic performances of real-time histologic prediction by digital chromoendoscopy

**Fig. 1:**
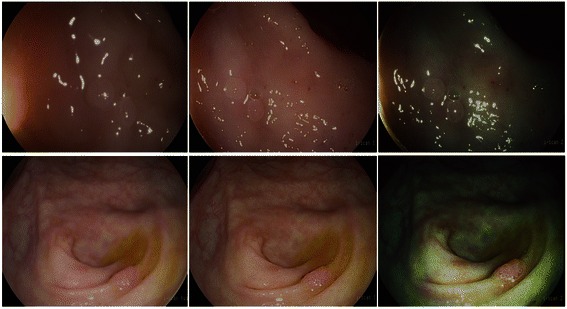
High definition white light and digital chromoendoscopy of hyperplastic (upper panels) and adenomatous diminutive polyps (lower panels). Each polyp was characterized according to size, shape and surface characteristics including pit pattern and mucosal vascular pattern morphology, with digital chromoendoscopy before resection. The distinction between hyperplastic (upper panels) and adenomatous polyps (lower panels) was made based upon previously published and validated criteria on the appearance of diminutive colonic polyps under digital chromoendoscopy [22]. Left panels: High definition white light; Middle panel: High definition i-scan 1 (default setting); Right panel: High definition i-scan 2 (default setting)

Each polyp was characterized according to size, shape and surface characteristics (pit pattern and mucosal vascular pattern morphology, colour, depression; Fig. 1) before resection and *in vivo* histology was predicted with a level of confidence (high or low). The endoscopic distinction between hyperplastic and adenomatous polyps was made based upon previously published and validated criteria as shown in Table 2 [22].

**Table 2:** Digital chromoendoscopy characteristics of diminutive colonic polyps [22]

	Hyperplasia	Adenoma
Mucosal pattern	No definite pits; circular or dotted pits	Oval, tubular, or elongated pits
Vascular pattern	No visualized vessels or exiguous thin superficial vessels	Short, thick vessels with distinct vascular density surrounding pits; overall increased vascular contrast compared to adjacent normal mucosa

Overall, the histology of 109 of the 121 polyps was predicted accurately through the real-time endoscopic appearance, leading to an accuracy of digital chromoendoscopy of 90.1 %. Of the 57 adenomas, only four were incorrectly predicted to be hyperplastic lesions, while 11 of the 63 non-adenomatous polyps were misdiagnosed as adenomas. As shown in Table 3, this equals a diagnostic sensitivity for real time assessment of 93.2 % (95 % CI, 82.7–97.8), a specificity of 88.7 % (95 % CI, 77.5–95) and a positive and negative prediction of 88.7 % (95 % CI, 77.5–95) and 93.2 % (95 % CI, 82.7–97.8), respectively. Intraobserver agreement was achieved in 113 out of 121 polyps (93.4 %), leading to an almost perfect κ coefficient of agreement of 0.867 [95 % CI: 0.799–0.967].

**Table 3:** Diagnostic performances for histology predictions by using digital chromoendoscopy

	Diagnostic performances				
	Polyps (n)	Sensitivity (95 % CI)	Specificity (95 % CI)	PPV (95 % CI)	NPV (95 % CI)
All distal polyps					
Overall prediction	121	93.3 % (82.7–97.8)	88.7 % (77.5–95)	88.7 % (77.5–95)	93.2 % (82.7–97.8)
HC prediction	107	98.1 % (88.6–99.9)	94.4 % (83.7–98.6)	94.5 % (83.9–98.6)	98.1 % (88.4–99.9)
Polyps in the rectosigmoid only					
Overall prediction	79	90.3 % (73.1–97.5)	87.5 % (74.1–94.8)	82.4 % (64.8–92.6)	93.3 % (80.1–98.3)
HC prediction	72	96.4 % (79.8–99.8)	95.5 % (83.3–99.2)	93.1 % (75.8–98.8)	97.7 % (86.2–99.9)

*CI* Confidence Interval, *PPV* Positive Predictive Value, *NPV* Negative Predictive Value, *HC* High Confidence

High confidence prediction of histology was made in 107 of 121 polyps (88.4 %). Considering only predictions made with high confidence, the accuracy of digital chromoendoscopy to predict histology was 96.3 % (103 out of 107). When only predictions made with high confidence were considered, diagnostic performances were markedly increased with a diagnostic sensitivity for adenoma prediction of 98.1 % (95 % CI, 88.6–99.9) and a specificity of 94.4 % (95 % CI, 83.7–98.6). Likewise, positive and negative predictive values were also increased to 94.5 % (95 % CI, 83.9–98.6) and 98.1 %, (95 % CI, 88.4–99.1) respectively, when predictions were made with high confidence. Of note, all 4 SSAs were predicted be to adenomatous lesions by digital chromoendoscopy, and predictions were made with high confidence in all SSAs.

Since most of the studies evaluating the real-time assessment of polyp histology included only polyps located in the sigmoid and rectum and in order to allow direct comparability between the studies, we performed a subgroup analysis in our study cohort on the diagnostic performance of digital chromoendoscopy for prediction of histology in diminutive polyps of the rectum and sigmoid only. 36.7 % (29 out of 79) of all polyps at this locale exhibited adenomatous histology while 63.3 % (50 out of 79) were hyperplastic lesions (Table 1).

When only diminutive polyps in the rectosigmoid were considered, digital chromoendoscopy allowed prediction of adenomatous histology with a sensitivity of 90.3 % (95 % CI, 73.1–97.5), a specificity of 87.5 % (95 % CI, 74.1–94.8), a positive predictive value of 82.4 % (95 % CI, 64.8–92.6) and a negative predictive value of 93.3 % (95 % CI, 80.1–98.3) (Table 3). Importantly, when only high confidence predictions in the rectosigmoid were considered, diagnostic performances were again markedly increased and exhibited values comparable to those observed for all distal polyps (including descending colon) in which predictions were made with high confidence. The following diagnostic performances were calculated for high confidence predictions of polyps in the rectosigmoid: sensitivity 96.4 % (95 % CI, 79.8–99.8), specificity 95.5 % (95 % CI, 83.3–99.2), and positive and negative prediction of 93.1 % (95 % CI, 75.8–98.8) and 97.7 % (95 % CI, 86.2–99.9), respectively.

### Prediction of future surveillance intervals

Based on recent European [19] and US guidelines [20], we compared the predicted surveillance intervals made with *in vivo* assessment by digital chromoendoscopy with those made by standard histopathology. This analysis was done on a per-patient level, and according to the requirements of the PIVI only patients in which a high confidence prediction of polyp histology was possible were considered for this analysis.

Considering all distal polyps, surveillance based on European guidelines was predicted correctly in 69 out of 73 patients (94.5 %) while agreement was achieved in 68 out of 73 patients (93.2 %) when using the US guidelines.

When analyzing surveillance intervals in diminutive polyps in the rectosigmoid only, an accurate calculation of surveillance intervals was possible in 96.4 % of the patients (53 out of 55) with both, US and European guidelines. Patients in whom surveillance intervals predicted with *in vivo* assessment by digital chromoendoscopy differed compared to intervals as determined by standard histopathology are shown in Table 4.

**Table 4:** Individual patients with discrepancies between predicted surveillance intervals made with *in vivo* assessment by digital chromoendoscopy and those made by standard histopathology

	Pathology based surveillance intervals (in years)		Endoscopy based surveillance intervals (in years)		Most advanced lesion at pathology
	*European*	*US*	*European*	*US*	
All distal polyps					
Patient 1	3	3	10	5 to 10	Advanced adenoma
Patient 2	3	3	10	5 to 10	Advanced adenoma
Patient 3	3	3	10	5 to 10	Advanced adenoma
Patient 4	3	3	10	5 to 10	Advanced adenoma
Patient 5	3	5	3	3	SSA
Rectosigmoid polyps					
Patient 1	3	3	10	5 to 10	Advanced adenoma
Patient 2	3	3	10	5 to 10	Advanced adenoma

SSA, sessile serrated adenoma

## Discussion

Here, we have shown that digital chromoendoscopy can reliable predict histology of distal diminutive polyps in real time according to the recommendations of the ASGE PIVI statement.

Distal diminutive colorectal polyps are common in the general screening population. Thus, an accurate endoscopic *in vivo* identification of polyps that are hyperplastic and therefore do not possess a risk of developing colorectal cancer is of major importance to reduce costs and risks associated with their redundant removal. Based on these considerations, the ASGE has proposed the PIVI statement on the real-time assessment of colorectal diminutive polyps [8] in which it is recommended that the technology used to guide the decision to leave a suspected hyperplastic polyp in the rectosigmoid in place should provide at least 90 % negative prediction for adenomatous histology. Further, in order for diminutive colorectal polyps to be resected and discarded without pathologic examination, a new technology should provide a ≥ 90 % agreement with histopathologic assessment in assigning post-polypectomy intervals.

Within this study we thus set off to prospectively evaluate digital chromoendoscopy for the real-time *in vivo* assessment of distal diminutive colorectal polyps and specifically questioned whether digital chromoendoscopy is sufficiently accurate (i) to allow distal hyperplastic polyps to be left in place without resection or (ii) to be resected and discarded without pathological assessment. We found that digital chromoendoscopy is indeed able to differentiate adenomatous from non-adenomatous histology in real time with a high overall sensitivity and specificity. Importantly, digital chromoendoscopy exhibited a negative prediction for ruling out adenomas of 98 % in high confidence predictions and predicted endoscopic surveillance intervals with ≥ 90 % agreement compared to histology based US and European surveillance guidelines, thereby exceeding by far the thresholds recommended by the ASGE for leaving suspected hyperplastic polyps in place and for resecting and discarding diminutive polyps without pathologic assessment [8]. Of note, these values included diminutive polyps ≤ 5 mm of the rectum, sigma and descending colon. When focusing on polyps of the rectum and sigmoid colon only, digital chromoendoscopy exhibited an equally high diagnostic performance for both, the negative prediction of adenomatous histology and the prediction of surveillance intervals.

The results of this study are consistent with previous studies on optical chromoendoscopy which have shown that NBI can predict polyp histology in real-time with high accuracy [14, 16, 24, 25]. Further, the available data illustrate that NBI indeed holds the potential to facilitate the management of distal diminutive polyps as it could accurately exclude adenomatous lesions with a sufficient negative prediction and also allowed for prediction of post polypectomy surveillance intervals in real time [13, 15, 26].

Recently, it has been demonstrated that digital chromoendoscopy is superior compared to standard white-light colonoscopy for the detection of colorectal neoplasms [27], and a prospective cohort study demonstrated that NBI and i-scan exhibit a similar efficiency for the histological prediction of diminutive polyps [22]. Further, analyzing polyps throughout the whole colon, it was shown that digital chromoendoscopy could predict histology in diminutive colorectal polyps with high accuracy and could also predict surveillance intervals [21]. However, in this report high definition white-light endoscopy exhibited the same high negative prediction for adenomatous histology as digital chromoendoscopy [21]. As already discussed by the authors, the results of this study might exhibit a certain bias, as the study followed a strict protocol of cleaning all polyps with water, simethicone, and N-acetylcysteine prior to the assessment of polyps [21].

Within this study, we included polyps of the distal colorectum and assessed them in real time under clinical conditions without pre-staining of the mucosa.

Importantly, our results show that the diagnostic performances of assessing histology are similar when all distal diminutive polyps were analyzed (including descending colon) or when the analysis was limited to diminutive polyps in the rectosigmoid only. These findings have important implications: Firstly, consistent with the results of the HiScope study [21], they provide further evidence that digital chromoendoscopy is indeed capable to translate the “leaving distal hyperplastic polyps in place” and “resect and discard” strategies into clinical practice, and secondly, they also allow a first insight that the “leaving in place” paradigm might not only be limited to polyps in the rectosigmoid, but could also be extended to the descending colon. However, the later aspect needs to be verified in a separate and large study cohort specifically addressing this question.

Although it has recently been suggested that SSAs may show changes in the microvasculature [28–30], their endoscopic identification and differentiation is challenging. Previous studies have shown that almost one third of all SSAs are misdiagnosed as hyperplastic polyps on NBI [31]. Since SSAs can progress to cancer through the serrated neoplasia pathway and can lead to sporadic microsatellite instability with a high colon cancer rate [32–34], their accurate endoscopic identification is of high clinical relevance. Although limited in number, all 4 SSAs included in the current study were correctly predicted be to adenomatous lesions by digital chromoendoscopy. Based on this, larger studies that assess whether digital chromoendoscopy can accurately predict SSA histology are highly warranted.

The strengths of this study are its prospective design and the assessment of a question that is highly relevant to daily endoscopic routine, and to which the answers are likely to influence and change daily practice. As a limitation, this study was conducted as a single center study and endoscopies were performed by a single endoscopist and it is anticipated that the interpretation of digital chromoendoscopy findings are also subject to inter-individual variations. Thus, studies in which less-experienced and community physicians perform digital chromoendoscopy to predict histology of diminutive polyps will be needed before this approach becomes widely clinically accepted. Nevertheless, digital chromoendoscopy is, similar to NBI, a simple “push-of-a-button” technology and data on NBI illustrate that the appearance of adenomatous polyps can be learned within 15 to 20 min with moderate inter- and substantial intraobserver agreement [35–37]. Similarly, endoscopists with varying levels of experience can accurately predict polyp histology using digital chromoendoscopy after a single training session [38, 39] and digital chromoendoscopy exhibits comparable accuracies for the detection of colonic lesions by non-expert and expert endoscopists [40].

Further, we did not include a separate control arm assessing whether high-definition endoscopy without the concomitant utilization of digital chromoendoscopy can adequately predict polyp histology. However, it has been shown recently that the adenoma detection rate does not differ between procedures utilizing high-definition or standard-definition endoscopes [41]. Further, it is known that high definition endoscopy in combination with digital chromoendoscopy is superior compared to white-light colonoscopy for the detection of colorectal neoplasms [27]. Based on these existing data, we thus aimed to address the remaining question whether high definition endoscopy in combination with digital chromoendoscopy is accurate to predict real time polyp histology, a question which can be well addressed with the design of our study. Nevertheless, it seems clear that in the future multi-center and multi-observer studies including experts and non-expert endoscopists should be performed to assess the inter-observer agreement and accuracy for predicting histology and also to assess the learning curve for predicting histology in diminutive colonic polyps using digital chromoendoscopic modalities. Further, it is important to demonstrate in the future that digital chromoendoscopy is as precise as optical chromoendoscopy and conventional dye based endoscopy.

## Conclusion

In summary, we have shown that digital chromoendoscopy allows an accurate real time prediction of the histology of diminutive polyps in the distal colon and by far exceed the thresholds recommended by the ASGE for a new technology to leave diminutive polyps in place or to resect and discard them without pathological assessment. Based on our results, further studies on the prediction of polyp histology with digital chromoendoscopy should be performed with the inclusion of less experienced physicians and polyps of the whole colon.

## Abbreviations

CRC: Colorectal cancer
ASGE: American Society for Gastrointestinal Endoscopy
PIVI: Preservation and incorporation of valuable endoscopic innovations
DLC: Dye-less chromoendoscopy
NBI: Narrow band imaging
CBI: Compound band imaging
FICE: Fuji intelligent color enhancement
SPIES: Storz professional image enhancement systems
CI: Confidence interval
PPV: Positive predictive value
NPV: Negative predictive value
HC: High confidence
